# Intensive care unit-acquired infection as a side effect of sedation

**DOI:** 10.1186/cc8907

**Published:** 2010-03-15

**Authors:** Saad Nseir, Demosthenes Makris, Daniel Mathieu, Alain Durocher, Charles-Hugo Marquette

**Affiliations:** 1 Intensive Care Unit, Calmette Hospital, University Hospital of Lille, boulevard du Pr Leclercq, 59037 Lille cedex, France; 2 Intensive Care Unit, University Hospital of Larisa, University of Thessaly, Biopolis Street, 41110 Larisa, Greece; 3 Respiratory Disease Department, University Hospital of Nice, Hôpital Pasteur, 30 avenue de la voie Romaine, BP 69, 06002 NICE cedex 1, France

## Abstract

**Introduction:**

Sedative and analgesic medications are routinely used in mechanically ventilated patients. The aim of this review is to discus epidemiologic data that suggest a relationship between infection and sedation, to review available data for the potential causes and pathophysiology of this relationship, and to identify potential preventive measures.

**Methods:**

Data for this review were identified through searches of PubMed, and from bibliographies of relevant articles.

**Results:**

Several epidemiologic studies suggested a link between sedation and ICU-acquired infection. Prolongation of exposure to risk factors for infection, microaspiration, gastrointestinal motility disturbances, microcirculatory effects are main mechanisms by which sedation may favour infection in critically ill patients. Furthermore, experimental evidence coming from studies both in humans and animals suggest that sedatives and analgesics present immunomodulatory properties that might alter the immunologic response to exogenous stimuli. Clinical studies comparing different sedative agents do not provide evidence to recommend the use of a particular agent to reduce ICU-acquired infection rate. However, sedation strategies aiming to reduce the duration of mechanical ventilation, such as daily interruption of sedatives or nursing-implementing sedation protocol, should be promoted. In addition, the use of short acting opioids, propofol, and dexmedetomidine is associated with shorter duration of mechanical ventilation and ICU stay, and might be helpful in reducing ICU-acquired infection rates.

**Conclusions:**

Prolongation of exposure to risk factors for infection, microaspiration, gastrointestinal motility disturbances, microcirculatory effects, and immunomodulatory effects are main mechanisms by which sedation may favour infection in critically ill patients. Future studies should compare the effect of different sedative agents, and the impact of progressive opioid discontinuation compared with abrupt discontinuation on ICU-acquired infection rates.

## Introduction

Healthcare-associated infections are the most common complications affecting hospitalized patients [1]. Intensive care unit (ICU)-acquired infections represent the majority of these infections [2]. In a recent multicenter study conducted in 71 adult ICUs [3], 7.4% of the 9,493 included patients had an ICU-acquired infection. ICU-acquired pneumonia (47%) and ICU-acquired bloodstream infection (37%) were the most frequently reported infections. Another recent multicenter study was conducted in 189 ICUs [4]. Of the 3,147 included patients, 12% had an ICU-acquired sepsis. ICU-acquired infections are frequently advocated as a significant contributor to mortality and morbidity [5,6]. Diagnosing these infections can be difficult in ICU patients with multiorgan failure. In addition, differentiating lower respiratory tract infection from colonization can be a difficult task in patients requiring mechanical ventilation [7]. Although mortality attributable to ICU-acquired infection is a matter of debate, high attributable morbidity and cost were repeatedly reported in patients with these infections [7-10].

Sedative and analgesic medications are routinely used in mechanically ventilated patients to reduce pain and anxiety and to allow patients to tolerate invasive procedures in the ICU [11]. Mostly a combination of an opioid, to provide analgesia, and a hypnotic, such as a benzodiazepine or propofol to provide anxiolysis, is used [12]. A variety of opioids used by intravenous administration in adults are available for use in the ICU, including morphine, fentanyl, alfentanil, sufentanil, and remifentanil [13-15].

Recently, several studies reported longer duration of mechanical ventilation and hospital stay in patients receiving sedation in the ICU [16,17]. Prolonged duration of mechanical ventilation and ICU stay are well-known risk factors for ICU-acquired infection. In addition, sedation could favour infection by several other mechanisms. The aim of this review is to discuss epidemiologic data that suggest a relation between infection and sedation, to review available data for the potential causes and pathophysiology of this relation, and to identify potential preventive measures.

## Materials and methods

Data for this review were identified through searches of PubMed, and from bibliographies of relevant articles. We undertook a comprehensive search in PubMed, from April 1969, through to April 2009, using the terms "infection AND sedation", "pneumonia AND sedation", "bloodstream infection AND sedation", "infection AND opioids", "infection AND hypnotics", or "infection AND opioid withdrawal" without time limit. The search was limited to publications in English and French.

Clinical studies were selected for this review if they reported on the relation between infection and sedatives used for long-term sedation in ICU patients. Animal and *in vitro *studies were included if they reported on the relation between infection and immunologic effects of sedation or on other potential mechanisms of infection in sedated patients. All abstracts were reviewed by two independent reviewers (SN and DeM). Articles of relevant abstracts were reviewed. All relevant articles were included in this review. After PubMed searches, 192 original articles were selected on abstracts. After reading these articles, 121 were kept in this review. Six additional original studies were found using references of selected articles.

## Results

### Epidemiology

Analgesia and sedation have routinely been employed in ICU patients for many years, particularly among those receiving mechanical ventilation. Surveys and prospective cohort studies have revealed wide variability in medication selection, monitoring using sedation scales and implementation of structured treatment algorithms among practitioners in different countries and regions of the world [18]. However, protocols that guide the clinician to administer the least necessary sedation to achieve patient comfort while maintaining patient-examiner interactivity are recommended [19]. In an international cohort study conducted in 1998 [17], 68% of the 5,183 mechanically ventilated adults received a sedative at any time while receiving mechanical ventilation. At least one analgesic or sedative drug was used on 58% of days of ventilatory support, including benzodiazepines in 69%, propofol in 21% and opioids in 63% of sedation days. Heterogeneity in clinical practice for different regions of the world was demonstrated, with use of analgesic and sedative drugs being most common in Europe and least common in Latin America. According to the results of a recent survey performed in 647 ICU physicians [20], substantial differences exist in sedative and analgesic practices in western European ICUs. Midazolam and propofol were the more frequently used sedatives, and morphine and fentanyl were the most frequently used analgesics. In France, a prospective, observational study was performed on 1,381 adult patients in 44 ICUs [21]. Sedatives were used less frequently than opioids (72% and 90%, respectively), and a large proportion of assessed patients (40 to 50%) were in a deep state of sedation.

In a retrospective case-control study, opiate analgesics were found to contribute to the development of postburn infectious complications when the burn injury is of a less severe nature [22]. With 187 controls, 187 patients with at least one infectious complication were matched according to age ± one year, length of hospital stay before infection, and total body surface area burned ± 5%. The median opiate equivalent was 14 in cases compared with 10 in controls (*P *= 0.06). Cases were more likely to be classified into the high opiate equivalent group relative to controls (odds ratio (OR), 1.24; 95% confidence interval (CI), 1 to 1.54; *P *= 0.049). The duration of opiate use was significantly longer in cases as compared with controls (*P *< 0.001). The association between opiate use and infection was modified by burn size. Limitations of this study included the retrospective observational design, and the absence of adjustment for comorbidities. In a large prospective observational multicenter study, an intermediate value (6 to 13) of the actual Glasgow coma scale on day 1, reflecting either preexisting disease or the effects of sedation, was significantly more frequent in patients with early-onset ventilator-associated pneumonia (VAP) compared with those without early-onset VAP (52% vs 37%, *P *= 0.03). In addition, a Glasgow coma scale value of 6 to 13 was independently associated with early-onset VAP (OR, 1.95; 95% CI, 1.2 to 3.18). In a prospective observational multicenter study, Metheny and colleagues determined risk factors for VAP [23]. A high level of sedation was identified as an independent risk factor for VAP (OR, 2.3; 95% CI, 1.3 to 4.1; *P *= 0.006). Other risk factors included abundant aspiration (OR, 4.2; 95% CI, 2.7 to 6.7; *P *< 0.001), and paralytic agent use (OR, 2.7; 95% CI, 1.6 to 4.5; *P *< 0.001).

Another recent prospective observational study evaluated risk factors for ICU-acquired infection [24]. Of the 587 patients, 39% developed at least one ICU-acquired infection. Although higher rates of sedation were found in patients with ICU-acquired infection compared with those without ICU-acquired infection (87% vs 53%; OR, 5.7; 95% CI, 3.7 to 8.9; *P *< 0.001), sedation was not independently associated with ICU-acquired infection. However, remifentanil withdrawal was identified as an independent risk factor for ICU-acquired infection (OR, 2.53; 95% CI, 1.28 to 4.19; *P *= 0.007). The highest rate of ICU-acquired infection was observed at day 4 after remifentanil discontinuation. However, this study was observational, and performed in a single center. Therefore, no cause-to-effect relation could be determined, and the results may not be applicable to patients hospitalized in other ICUs. Results of studies reporting on the relation between sedation and ICU-acquired infection are presented in Table 1.

**Table 1:** Results of studies reporting on relation between sedation and infection

First author [Reference]	Year of publication/country	Setting	Study design/Number of patients	Type of infection	Number of patients with sedation				
						
					Type of sedation	Infection	Number of infections	*P*	OR (95% CI)
Bornstain [29]	2004/France	Mixed ICUs	Prospective cohort/747	Early-onset VAP	NR*	42/80 (52)	251/667 (37)	0.03	1.9 (1.2-3.1)**
Schwacha [22]	2006/USA	Burn unit	Retrospective nested case-control study/374	Hospital-acquired infection	Opiate analgesics	NR	NR	0.049^§^	1.2 (1-1.5)
Metheny [23]	2006/USA	Mixed ICUs	Prospective cohort/360	VAP	NR	150/173 (86)	132/187 (70)	0.006	2.3 (1.3-4.1)**
Nseir [24]	2009/France	Mixed ICU	Prospective cohort/587	ICU-acquired infection	Remifentanil with or without midazolam	203/233 (87)	191/354 (53)	<0.001	5.7 (3.7-8.9)

*Results for patients with neurologic impairment at ICU admission, the number of patients with neurologic impairment related to sedation or to preexisting disease was not reported.

**Adjusted odds ratio (OR).

^§^*P *value for the difference in rate of cases and controls classified into the high opiate equivalent group.

CI: confidence interval; ICU: intensive care unit; NR: not reported; VAP: ventilator-associated pneumonia;

The data from these epidemiologic studies suggest that there is a potential association between sedation and infection. In light of the wide and variable application of sedatives in ICU patients, where management of infection is crucial, the relation between sedative agents and infection merits further investigation.

### Pathophysiology

#### Exposure to risk factors for ICU-acquired infection

Several studies demonstrated that sedation prolongs exposure to risk factors for ICU-acquired infection. In a prospective observational cohort study performed on 252 consecutive ICU patients requiring mechanical ventilation [16], Kollef and colleagues found that duration of mechanical ventilation was significantly longer for patients receiving continuous intravenous sedation compared with patients not receiving continuous intravenous sedation (185 ± 190 vs 55.6 ± 75.6 hours; *P *< 0.001). Similarly, the lengths of intensive care (13.5 ± 33.7 vs 4.8 ± 4.1 days; *P *< 0.001) and hospitalization (21.0 ± 25.1 vs 12.8 ± 14.1 days; *P *< 0.001) were statistically longer among patients receiving continuous intravenous sedation. In a multicenter study performed on a cohort of 5,183 patients receiving mechanical ventilation [17], a total of 3,540 (68%) patients received sedation. The persistent use of sedatives was associated with more days of mechanical ventilation (median, 4 (interquartile range (IQR), 2 to 8), vs 3 (2 to 4) days, *P *< 0.001; in patients who received sedatives, and those who did not receive sedatives; respectively); and longer length of stay in the ICU (8 (5 to 15), vs 5 (3 to 9) days, *P *< 0.001). Further, muscle relaxants are adjuncts to sedation in some patients. The use of muscle relaxant agents is a well-known risk factor for polyneuropathy and prolonged mechanical ventilation duration [18].

Duration of mechanical ventilation is a well-known risk factor for VAP. Cook and colleagues [25] reported that the cumulative risk of VAP increased over time, although the daily hazard risk decreased after day 5 of mechanical ventilation (3.3% at day 5, 2.3% at day 10, and 1.3% at day 15). Prolonged stay in the ICU is associated with increased exposure to invasive procedures such as intubation, and central venous, arterial and urinary catheters. Device use is the major risk factor for VAP, bloodstream infection, and urinary tract infection [3,26,27].

#### Microaspiration

Many studies have found an association between coma as the reason for ICU admission and VAP [25,28-31]. One potential explanation for the association between neurologic impairment and VAP is microaspiration of contaminated oropharyngeal secretions. Bacterial colonization of the aerodigestive tract and entry of contaminated secretions into the lower respiratory tract are critical in the pathogenesis of VAP [32]. The endotracheal tube is an important risk factor for VAP, because it permits leakage of oropharyngeal secretions around the cuff and may act as a nidus for the growth of intraluminal biofilms [33]. A recent prospective observational study aimed to determine the frequency of pepsin-positive tracheal secretions (a proxy for the aspiration of gastric contents), outcomes associated with aspiration, and risk factors for aspiration in 360 critically ill tube-fed patients [23]. Almost 6,000 tracheal secretions collected during routine suctioning were assayed for pepsin; of these, 31.3% were positive. At least one aspiration event was identified in 88.9% (n = 320) of the participants. The incidence of pneumonia (as determined by the Clinical Pulmonary Infection Score) increased from 24% on day 1 to 48% on day 4. Patients with pneumonia on day 4 had a significantly higher percentage of pepsin-positive tracheal secretions than did those without pneumonia (42.2% vs. 21.1%, respectively; *P *< 0.001). Interestingly, a Glasgow Coma Scale score of less than nine (*P *= 0.021) was significantly associated with aspiration by univariate analysis. Other risk factors for aspiration included a low backrest elevation (*P *= 0.024), vomiting (*P *= 0.007), gastric feedings (*P *= 0.009), and gastroesophageal reflux disease (*P *= 0.033). In a 24-hour manometric study, esophageal motility was investigated in 21 adults, including 15 consecutive ventilated patients, and 6 healthy volunteers [34]. Irrespective of the underlying disease, propulsive motility of the esophageal body was significantly reduced during any kind of sedation. Impaired tubular esophageal motility is involved in the pathogenesis of gastrointestinal reflux disease, which, in turn has been shown to cause nosocomial pneumonia in critically ill patients.

### Microcirculatory effects of sedation

In a pilot study performed on 10 ICU patients, benzodiazepine induced an increase in cutaneous blood flow secondary to vasodilation, a decrease in reactive hyperemia, and alterations of vasomotion [35]. Addition of sufentanil did not substantially modify the results obtained. Clinical studies have clearly established that alterations of normal microcirculatory control mechanisms may compromise the tissue nutrient blood flow and may contribute to the development of organ failure in septic patients [36,37]. In addition, numerous experimental studies have reported that microvascular blood flow is altered in sepsis and common findings include a decrease in functional capillary density and heterogeneity of blood flow with perfused capillaries in close vicinity for nonperfused capillaries [38,39]. Multiple factors may contribute to these findings, including alterations in red blood cell rheology and leucocyte adhesion to endothelial cells, endothelium dysfunction, and interstitial edema. These observations suggest that sedation may alter tissue perfusion when already compromised, as in septic patients, and contribute to the development of multiorgan failure.

### Intestinal effects of sedation

Gastrointestinal motility disturbances are common in critically ill patients [40]. These disturbances cause considerable discomfort to the patients and they are also associated with an increased rate of complications. In addition, fecal stasis induces microbiological imbalance, resulting in overgrowth of Gram-negative bacteria, relative reduction of the endogenous anaerobic and Gram-positive flora, and increase in endotoxin load. Translocation of bacteria may lead to infections, and translocation of endotoxins may enhance systemic inflammation [41-44]. Opioid drugs inhibit gastrointestinal transit by inhibiting neurotransmitter release and by changing neural excitability [45]. An animal model demonstrated that one-quarter of the dose needed to produce analgesia inhibits intestinal motility and one-twentieth of the analgesic dose is sufficient to stop diarrhea [40]. In contrast to many other opioid-induced side effects such as nausea, vomiting, and sedation, patients rarely develop tolerance to constipating effects of opioids [46]. Dexmedetomidine was also found to inhibit gastric, small bowel, and colonic motility [47]. In contrast, continuous infusion of propofol does not alter gastrointestinal tract motility more than a standard isolflurane anaesthesia [48].

### Immunomodulatory effects of sedation

#### Opioids

Experimental evidence coming from *in vitro *and *in vivo *animal studies suggests that opioids may alter the immunologic response to exogenous stimuli resulting in higher risk of infection. Opioids have been found to have deleterious effects on host immunity across a broad range of pathogenic microorganism [49-55]. Their immunomodulatory effects have been observed following acute and chronic exposure and after opioid withdrawal in several infectious models.

##### 1. Acute exposure to opioids

Acute exposure to morphine suppresses mitogen-stimulated proliferation of T- and B-lymphocytes [56,57], natural killer (NK) cell cytotoxic activity, primary antibody production [58-60], phagocytosis by macrophages [61,62], macrophage migration via its apoptotic effects [63], and IL2, interferon γ (IFN), TNF-α, and nitric oxide (NO) production [64-71]. These suppressive effects are blocked by naloxone, a competitive opioid antagonist, suggesting that the effects are mediated via opioid receptors [72]. Location of opioid receptors on immunocytes suggests that morphine suppressive effects on the immune system may be due to a direct interaction [73-76]. Another possible mechanism is that central opioid receptors activate the sympathic nervous system and the hypothalamic-pituitary-adrenal axis, which subsequently suppress immune function [77-80]. The production of cathecolamines and neuropeptides from sympathic nerves and glucocorticoids from the adrenals are responsible for many of the immunomodulatory effects of morphine [81]. Recently, the neuroimmune mechanism of opioid-mediated conditioned immunomodulation was investigated [81-84]. Saurer and colleagues [83] provided evidence that the expression of morphine conditioned effects on NK cell activity requires the activation of dopamine D1 receptors in the nucleus accumbens shell. Furthermore, the antagonism of NPY Y1 receptor prevents the conditioned suppression of NK activity, suggesting that the conditioned and unconditioned effects of morphine involve similar mechanisms. Zaborina and colleagues [85] demonstrated that *Pseudomonas aeruginosa *can intercept opioid compounds released during host stress and integrate them into core elements of quorum sensing circuitry leading to enhanced virulence. These authors found that κ-opioid receptor agonists induce pyocyanin production in *P. aeruginosa*, and that dynorphin is released into the intestinal lumen following ischemia/reperfusion injury and accumulates in desquamated epithelium, where it binds to *P. aeruginosa*. Wang and colleagues [86] found that morphine treatment impairs TLR9-NF-κB signalling and diminishes bacterial clearance following *Streptococcus pneumoniae *infection in resident macrophages during the early stages of infection, leading to a compromised innate immune response. Another suggested mechanism for the immunosuppressive effects of morphine is enhancement of cellular apoptosis. In an *in vitro *study performed on lymphocytes infected with simian immunodeficiency virus (SIV), morphine-induced alteration in apoptotic and anti-apoptotic elements was found to be associated with accelerated viral progression [87]. One could wonder whether the immunomodulatory effects of sedative agents could be beneficial in septic patients by damping down an uncontrolled immune response to sepsis. However, to our knowledge, no published data support this hypothesis.

##### 2. Chronic exposure to opioids

Morphine immunopharmacological effects following chronic administration are controversial. Kumar and colleagues [88] reported that chronic morphine exposure caused pronounced virus replication in the cerebral compartment and accelerated onset of AIDS in SIV/SHIV-infected Indian rhesus macaques. Moreover, chronic exposure to morphine altered lipopolysaccharide (LPS)-induced inflammatory response and accelerated progression to septic shock in the rat [89]. Martucci and colleagues [90] analyzed the effects of fentanyl and buprenophine on splenic cellular immune responses in the mouse. They found that opioid-induced immunosuppression was less relevant in chronic administration than in acute or short-time administration. In mice implanted with morphine pellets, concanavalin (Con) A and LPS-stimulated splenocyte proliferation is maximally suppressed at 72 hours post implantation [91]. This suppression recovered by 96 hours independent of plasma morphine concentration, suggesting tolerance development [92]. Another study reported tolerance to morphine-induced suppression of NK cell activity after a 14 day period of chronic morphine administration [93]. Avila and colleagues [94] found that animals chronically treated with morphine became tolerant to its effects on the hypothalamic-pituitary-adrenal axis, and to its effects on T-lymphocyte proliferation. In contrast, other studies report that immune status does not recover after chronic morphine administration [60,95,96].

##### 3. Opioid withdrawal

Several recent animal studies reported profound and prolonged immunosuppressive effects during the period following opioid withdrawal. Increased levels of corticosterone were observed on sudden withdrawal of morphine administration [94,97], with return to basal levels within 72 hours. A significant suppression of lymphocyte responses was also observed within 24 hours after cessation of morphine administration. The suppression of lymphocyte proliferation was significant up to 72 hours of withdrawal of chronic morphine [94]. A decrease in animal weight, with a peak occurring at 24 hours following withdrawal induction, and a time-dependent suppression of concalavalin A (Con-A) and toxic shock syndrome toxin (TSST)-1-stimulated splenic T-cell proliferation, Con-A-stimulated splenocyte, IFN-γ production, and splenic NK cell activity were also reported [98]. Because clonidine inhibited these norepinephrine-dependent systems, it was suggested that opioid withdrawal-induced hyperactivity of the sympathic nervous system, and hypothalamic-pituitary-adrenal axis were responsible for these immunomodulatory effects. Abrupt morphine withdrawal, by removal of morphine pellets from dependent animals, resulted in profound immunosuppression that was maximal at 48 hours after pellet removal and was still present at 144 hours. In contrast, precipitated withdrawal, by removal of morphine pellets from dependent animals and injection of opioid antagonist, resulted in a short period of immunopotentiation at three hours after pellet removal, followed by profound immunosuppression at 24 hours post-withdrawal with a rapid return to normal immune response by 72 hours [99]. In an *in vitro *model, morphine withdrawal enhances HIV infection of peripheral blood lymphocytes and T cell lines through the induction of substance P [100]. Further, morphine withdrawal favoured hepatitis C virus (HCV) persistence in hepatic cells by suppressing IFN-α-mediated intracellular innate immunity and contributed to the development of chronic HCV infection [101]. Other studies, performed in mice, demonstrated that morphine withdrawal was associated with increased production of TNF-α and NO, and decreased IL-12 levels [102,103]. Feng and colleagues [104] showed that morphine withdrawal sensitizes to oral infection with a bacterial pathogen and predisposes mice to bacterial sepsis. Withdrawal significantly decreased the mean survival time and significantly increased the Salmonella burden in various tissues of infected mice compared with placebo-withdrawn animals. Increased bacterial colonization in this variety of tissues was observed from one day to as long as six days after withdrawal.

#### Benzodiazepines

It was suggested that benzodiazepines bind to specific receptors on macrophages and inhibit their capacity to produce IL-1, IL-6, and TNF-α [105]. Several studies have found that midazolam inhibits human neutrophil function and the activation of mast cells induced by TNF-α *in vitro *and suppresses the expression of IL-6 mRNA in blood monoclear cells [106]. Midazolam and propofol were found to inhibit both chemotaxis and exocytosis of mast cells, whereas thiopental only inhibited chemotaxis, and ketamine only inhibited exocytosis [107]. *In utero *exposure of rats to low dosages of diazepam has been found to result in depression of cellular and humoral immune responses during adulthood, with marked changes in macrophage spreading and phagocytosis. An impaired defence against *Mycobacterium bovis *was found in adult hamsters after *in utero *exposure to a dosage of 1.5 mg/kg of diazepam [108]. These effects could be explained by a direct and/or indirect action of diazepam on the cytokine network. They could also be related to stimulation of peripheral benzodiazepine receptor binding sites (PBR) by macrophages and/or lymphocytes, or they may be mediated by PBR stimulation of the adrenals [109]. In contrast, other investigators reported that midazolam did not alter LPS-stimulated cytokine response *in vitro*, or cytokine production in septic patients [110,111].

#### Propofol

An *in vitro *study tested the effects of propofol and midazolam on neutrophil function during sepsis [112]. In both early (at 4 hours) and late (at 24 hours) sepsis, propofol and midazolam depressed hydrogen peroxide production by blood and peritoneal neutrophils at clinical concentrations. Propofol caused more depression than midazolm (*P *< 0.005). Further, propofol was found to improve endothelial dysfunction and to attenuate vascular superoxide production in septic rats [113]. Propofol treatment attenuated the overproduction of NO and superoxide, thus restoring the acetylcholine-responsive NO-cyclic guanosine monophosphate (GMP) pathway in cecal ligation and puncture (CLP)-induced sepsis. It also significantly improved the CLP-impaired endothelium-dependent relaxation and endothelium-derived NO in a parallel manner. In rats with endotoxin-induced shock, treatment with propofol suppressed the release of TNF-α, IL-1β, IL-10, and NO production [114]. In addition, in anesthetized rabbits with acute lung injury, propofol attenuated lung leucosequestration, pulmonary edema, pulmonary hyperpermeability, and resulted in better oxygenation, lung mechanics, and histologic changes [115]. Taken together, these findings suggest that propofol administration could be beneficial in sepsis.

#### Clonidine and dexmedetomidine

Studies have shown that central-acting alpha-2 agonists inhibit noradrenergic neurotransmission and have a strong sedative component secondary to sympathetic inhibition [116]. This formerly adverse side effect is widely used nowadays in critical care settings to sedate patients and to reduce the amount of co-medication needed. A recent study has shown the beneficial effects of dexmedetomidine over lorazepam as an adjunct sedative in a critical care setting [117]. Furthermore, clonidine is an integral part of the sedation regimen in German ICUs [118].

Evidence that the clinically used medication clonidine has the potential to be a prophylactic option in treating sepsis has come from Kim and Hahn [119]. They have shown that clonidine pre-medication is able to significantly reduce the pro-inflammatory cytokines IL-1β and IL-6 in patients undergoing hysterectomy.

In rats, with endotoxin-induced shock, dexmedetomidine dose-dependently attenuated extremely high mortality rates and increased plasma cytokine concentration [120]. In addition, the early administration of dexmedetomidine drastically reduced mortality and inhibited cytokine response in endotoxin-exposed rats. Moreover, Hofer and colleagues [121] demonstrated that clonidine and dexmedetomidine improve survival in murine experimental sepsis. Down-regulation of pro-inflammatory mediators due to sympatholytic effects of the above mentioned drugs most probably responsible for this effect. The authors suggested that sympatholytics such as clonidine or dexmedetomidine may therefore be useful adjunct sedatives in the pre-emptive treatment of patients with a high risk for developing sepsis. However, recent studies ruled out a cholinergic interaction between the vagus nerve and the immune system [122]. Physiologic studies understanding the neuroimmune connections can provide major advantages to design novel therapeutic strategies for sepsis [123].

#### Barbiturates

Barbiturates are used for deep sedation in patients with elevated intracranial pressure refractory to standard therapeutic regimens. Correa-Sales and colleagues [124] showed that antigen-specific lymphocyte proliferation and IL-2 production by peripheral blood lymphocytes from patients under thiopental anesthesia were significantly depressed. In contrast, mitogen-induced lymphocyte proliferation, IL-2, and IL-4 secretion were not depressed. In spite of the transient decrease in antigen-driven IL-2 synthesis, no clinical evidence of infection was noted in any healthy patient. In an *in vivo *study, pentobarbital suppressed the expression of TNF-α mRNA and its proteins, which may result from a decrease in the activities of nuclear factor-κB and activator protein 1 and the reduction of the expression of p38 mitogen-activated protein kinase by pentobarbital [125]. In addition, pentobarbital directly enhanced the viabilities of cells, and protected cells from apoptosis induced by deferoxamine mesylate-induced hypoxia. Further, in an *in vitro *model substantially different effects of barbiturates and propofol were found on phagocytosis of *Staphylococcus aureus *[126]. The inhibitory effects of barbiturates demonstrated a strong dose-dependency. Impairment of phagocytosis activity was more pronounced than granulocyte recruitment.

**Figure 1:**
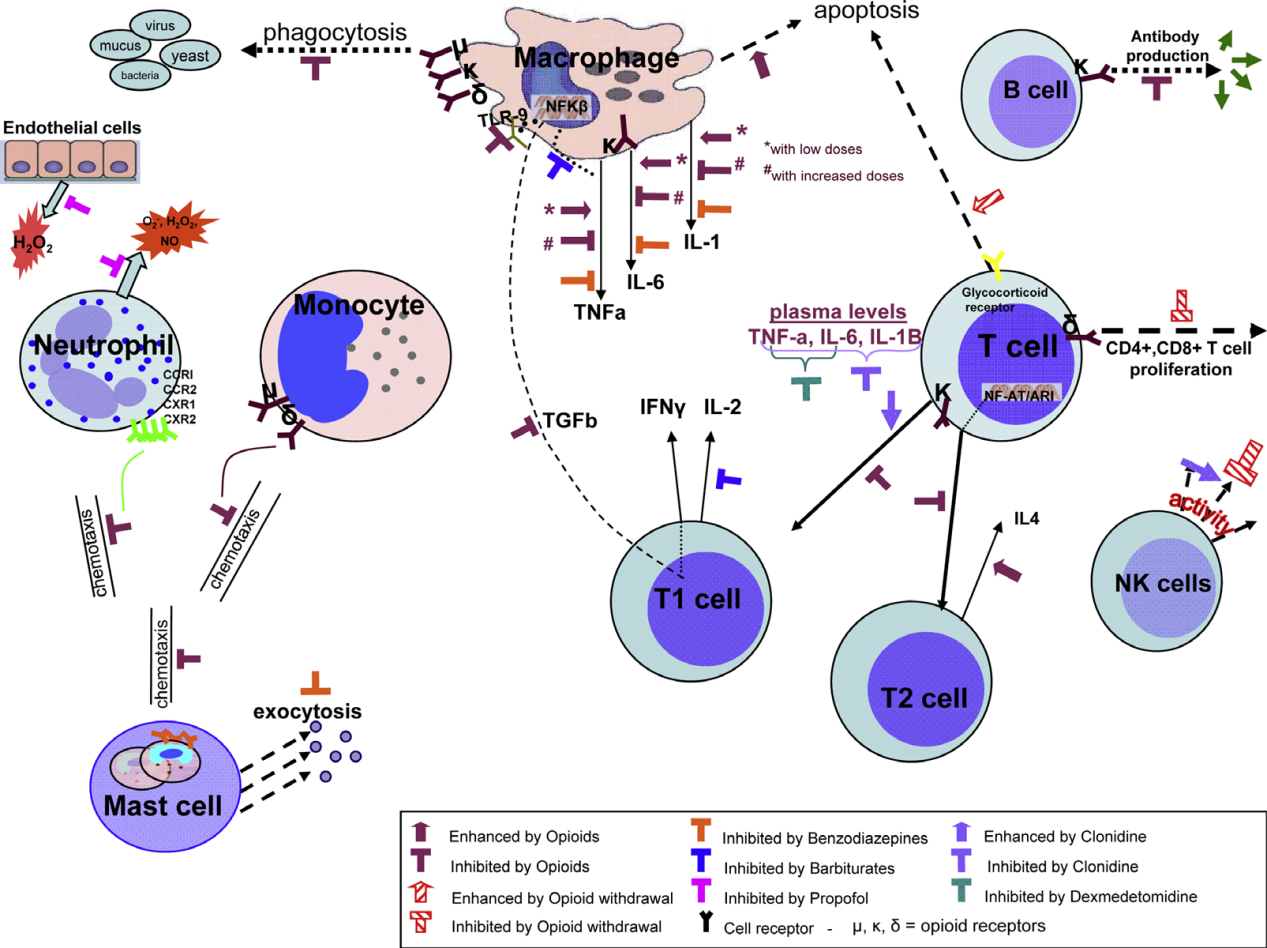
**Potential mechanisms of immunomodulatory effects of sedative agents**.

**Figure 2:**
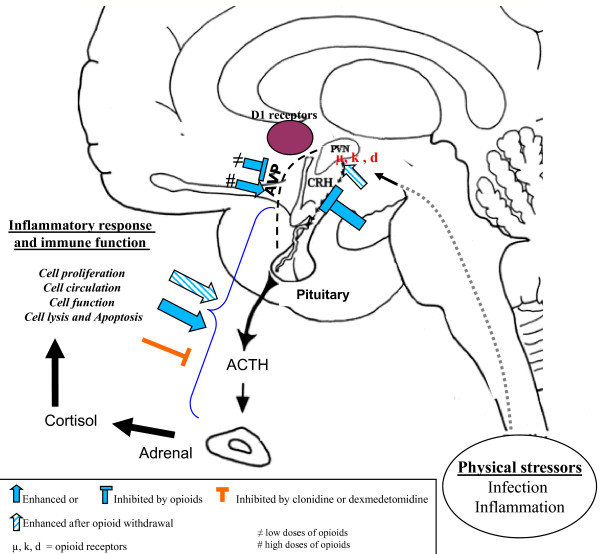
**Neuroimmune effects of sedative agents**.

Mechanisms by which sedation might favor infection are presented in Tables 2 and 3, and Figures 1 and 2.

**Table 2:** Mechanisms by which sedation might promote ICU-acquired infection

Mechanism	References	Study design/Number of patients	Main results
Prolongation of exposure to risk factors			
Longer duration of mechanical ventilation, and ICU stay	[17,23]	Prospective cohorts/5183, and 252; respectively	Durations of mechanical ventilation and ICU stay significantly longer in patients receiving sedation compared with those without sedation
Microaspiration			
Neurologic impairment	[23]	Prospective cohort/360	Heavy sedation significantly associated with microaspiration confirmed by pepsin-positive tracheal aspirate
Impaired tubular esophageal motility	[34]	Prospective cohort/21	Esophageal motility significantly reduced in sedated patients compared to healthy controls
Microcirculatory disturbances	[35]	Prospective cohort/10	Sedation induced an increase in cutaneous blood flow, a decrease in reactive hyperemia, and alterations of vasomotions
Gastrointestinal motility disturbances			
Opioids	[40]	Double-blind, placebo-controlled, randomized study comparing the effects of lactulose, polyethylene glycol, or placebo on defecation/308	Morphine administration associated with a longer time before first defecation, except in the polyethylene glycol group
Dexmedetomidine and clonidine	[47]	Animal study/NA	Clonidine and dexmedetomidine concentration-dependently increased peristaltic pressure threshold and inhibited peristalsis
Immunomodulatory effects	-	-	Please see Table 3 for details

ICU: intensive care unit; NA: not applicable.

**Table 3:** Immunomodulatory effects of sedative agents used in ICU patients

Sedative agent	References	Main results
Opioids	[55,56,99]	Suppression of mitogen-stimulated proliferation of T and B-lymphocytes
	[57-59,97]	Suppression of natural killer, and primary antibody production
	[60-62]	Inhibition of phagocytosis by macrophages
	[63-70,101,102]	Suppression of IL2, IL12, INFγ, and NO production
	[77-80,82,83,94,97-99]	Activation of sympathic nervous system, and the hypothalamic-pituitary-adrenal axis
	[84]	Enhancement of *Pseudomonas aeruginosa *virulence
	[85]	Reduction of bacterial clearance via impairment of TLR9-NF-κB signaling
	[86]	Enhancement of cellular apoptosis
Benzodiazepines	[105]	Inhibition of IL-1, IL-6, and TNF-α production
	[109]	Supression of macrophage migration and phagocytosis
Clonidine and dexmetetomidine	[119]	Reduction of IL-1β, and IL6 production
	[121]	Sympatholytic effects
Propofol	[112,113]	Suppression of H_2_O_2_, NO, and O* production; improvement of endothelial dysfunction
	[113]	Suppression of TNF-α, IL-β, IL-10
	[114]	Attenuation of leukosequestration, pulmonary edema, and pulmonary hyperpermeability
Barbiturates	[124]	Suppression of antigen-specific lymphocyte proliferation, and IL-2 production
	[125]	Suppression of TNF-α mRNA expression
	[126]	Impairment of phagocytosis

ICU: intensive care unit; IL: interleukin; INF: interferon; NO: nitric oxide; TNF: tumor necrosis factor.

## Discussion

### Modulation of sedation to prevent ICU-acquired infection

#### Daily interruption of continuous sedation

Recently, the impact of daily interruption of continuous sedative infusions on patient outcome was evaluated by a randomized controlled trial involving 128 adult patients receiving continuous sedation and mechanical ventilation in a medical ICU [127]. Duration of mechanical ventilation was significantly shorter in the daily interruption group compared with control group (median 4.9 vs 7.3 days, *P *= 0.004). Complications related to undersedation, such as removal of the endotracheal tube by the patient, were similar in the two groups. These results were confirmed by two subsequent randomized trials that paired daily interruption of sedation with ventilator weaning protocol [128], or early physical and occupational therapy [129]. Several recent studies evaluated the efficacy of an expanded ventilator bundle, including daily interruption of sedation, for the reduction of VAP in ICU patients [130-135]. A significant reduction of VAP rate was found by these studies. However, many of these studies are difficult to interpret because they do not report bundle compliance rate, do not control for other specific VAP risk factors, and use the clinical definition of VAP [136]. In addition, whether this reduction in VAP rate is related to daily interruption of sedation or to other measures used to prevent VAP, such as head-of-bed-elevation, peptic ulcer disease prophylaxis, oral care, or hand washing, is unknown.

#### Nurse-implemented sedation protocol

In a randomized controlled trial including 321 patients [137], Brook and colleagues compared a practice of protocol-directed sedation during mechanical ventilation implemented by nurses with traditional non-protocol-directed sedation administration. The median duration of mechanical ventilation was significantly shorter in patients managed with protocol-directed sedation compared with patients receiving non-protocol-directed sedation (55.9 vs 117 hours, *P *= 0.008). Lengths of stay in the intensive care unit (5.7 ± 5.9 vs 7.5 ± 6.5 days; *P *= 0.013) and hospital (14.0 ± 17.3 vs 19.9 ± 24.2 days; *P *< 0.001) were also significantly shorter among patients in the protocol-directed sedation group. In addition, a before-and-after prospective study found the implementation of a nursing-driven protocol of sedation to be associated improved probability of successful extubation in a heterogeneous population of mechanically ventilated patients [138]. Another recent randomized study compared daily interruption of sedation and sedation algorithms in 74 patients under mechanical ventilation [139]. The protocol-directed sedation group had shorter duration of mechanical ventilation (median 3.9 vs 6.7 days; *P *= 0.0003), faster improvement of Sequential Organ Failure Assessment over time (0.23 vs 0.7 units per day; *P *= 0.025), shorter ICU length of stay (8 versus 15 days; *P *< 0.0001), and shorter hospital length of stay (12 vs 23 days; *P *= 0.01). However, two recent Australian trials provided no evidence of a substantial reduction in the duration of mechanical ventilation or length of stay with the use of protocol-directed sedation compared with usual local management [140,141]. Qualified high-intensity nurse staffing and routine Australian ICU nursing responsibility for many aspects of ventilatory practice may explain the contrast between these findings and other studies.

Quenot and colleagues [142] performed a prospective before-after study to determine the impact of a nurse-implemented sedation protocol on the incidence of VAP. A total of 423 patients were enrolled (control group, n = 226; protocol group, n = 197). The incidence of VAP was significantly lower in the protocol group compared with the control group (6% and 15%, respectively; *P *= 0.005). A nurse-implemented protocol was found to be independently associated with a lower incidence of VAP after adjustment on Simplified Acute Physiology Score II in the multivariate Cox proportional hazards model (hazard rate, 0.81; 95% CI, 0.62 to 0.95; *P *= 0.03). The median duration of mechanical ventilation was significantly shorter in the protocol group compared with the control group (4.2 vs 8 days; *P *= 0.001). Potential means to reduce ICU-acquired infection in sedated patients are presented in Table 4.

**Table 4:** Potential means to reduce ICU-acquired infection in sedated patients

Intervention	First author [Reference]	Year of publication/country	Study design/Number of patients	Main results*
Daily interruption of sedation	Kress [127]	2000/USA	Randomized controlled/128	Shorter duration of MV (median 4.9 vs 7.3 d, *P *= 0.004)
Daily interruption of sedation, and ventilator weaning protocol	Girard [128]	2008/USA	Randomized controlled/336	Higher number of MV-free days (14.7 vs 11.6 days; *P *= 0.02) Shorter mean duration of ICU stay (9.1 vs 12.9 days; *P *= 0.01) Reduced ICU mortality (HR 0.68, 95% CI 0.5 to 0.92; *P *= 0.01)
Daily interruption of sedation, and early physical therapy	Schweickert [129]	2009/USA	Randomized controlled/104	Higher number of MV-free days (23 vs 21 days, *P *= 0.05) Higher rate of hospital discharge (59% vs 35%, *P *= 0.02)
Expanded ventilator bundle, including daily interruption of sedation	Papadimos [130]	2008/USA	Before-after cohort/2968	Reduced incidence rate of VAP (7.3 vs 19.3/1000 MV-days, *P *= 0.028)
	Blamoun [131]	2009/USA	Before-after cohort/NR	Reduced incidence rate of VAP (0 vs 12/1000 MV-days, *P *= 0.0006)
	Resar [132]	2005/USA and Canada	Before-after cohort/NR	Reduced incidence rate of VAP (2.7 vs 6.6/1000 MV-days)
	Berriel-Cass [133]	2006/USA	Before-after cohort/NR	Reduced incidence rate of VAP (3.3 vs 8.2/1000 MV-days)
	Youngquist [134]	2007/USA	Before-after cohort/NR	Reduced incidence rate of VAP (2.7 vs 6; and 0 vs 2.6/1000 MV-days)
	Unahalekhaka [135]	2007/Thailand	Before-after cohort/NR	Reduced incidence rate of VAP (8.3 vs 13.3/1000 MV-days)
Nurse-implemented sedation protocol	Brook [137]	1999/USA	Randomized controlled/321	Shorter duration of MV (55.9 vs 117.0 hours, *P *= 0.008) Shorter length of ICU stay (5.7 ± 5.9 vs. 7.5 ± 6.5 days; *P *= 0.013)
	Arias-Rivera [138]	2008/Spain	Before-after cohort/356	Increased rate of successful extubation (*P *= 0.002)
	Quenot [142]	2007/France	Before-after cohort/423	Reduced incidence of VAP (6 vs 15%, *P *= 0.005) Shorter duration of MV (4.2 vs 8 days, *P *= 0.001)

*intervention group compared with control group, respectively.

CI: confidence interval; HR: hazard ratio; ICU: intensive care unit; MV: mechanical ventilation; NR: not reported; VAP: ventilator-associated pneumonia;

#### Comparison of sedative agents

In a prospective randomized pilot study, the influence of fentanyl-based versus remifentanil-based anesthesia on cytokine responses and expression of the suppressor of cytokine signalling (SOCS)-3 gene was compared in 40 patients following coronary artery bypass graft surgery [143]. The IFN-γ/IL-10 ratio after Con-A stimulation in whole blood cells on post-operative day 1, and SOCS-3 gene expression on post-operative day 2 were significantly lower in the remifentanil group than in the fentanyl group. The time in the ICU was also significantly lower in the remifentanil group. These findings suggest that remifentanil can attenuate the exaggerated inflammatory response that occurs after cardiac surgery with cardiopulmonary bypass. Two recent randomized controlled studies found a remifentanil/propofol-based sedation regimen to be associated with shorter duration of mechanical ventilation and ICU stay compared with a conventional regimen [14,15].

In a double-blind randomized placebo-controlled trial performed in 33 newborn babies, sedation provided by continuous infusion of midazolam and morphine was comparable to morphine alone, with no significant adverse effects [144]. Interestingly, infection rate was similar in the two groups. The effects of prolonged infusion of midazolam and propofol on immune function were compared in a randomized study including 40 critically ill surgical patients who were to receive long-term sedation for more than two days [145]. Although midazolam suppressed the production of the pro-inflammatory cytokines IL-1β, IL-6 and TNF-α, both agents caused suppression of IL-8 production. Propofol inhibited IL-2 production and stimulated IFN-γ production, whereas midazolam failed to do so. Kress and colleagues [146] compared propofol and midazolam in a randomized study involving 73 patients (37 in propofol group and 36 in midazolam group). The propofol group had a significantly narrower range of wake-up times with a higher likelihood of waking in less than 60 minutes.

An observational study found patients with withdrawal syndrome to have significantly elevated hemodynamic, metabolic, and respiratory demands [147]. Clonidine significantly decreased these demands, induced mild sedation, and facilitated patient cooperation with the ventilator, enabling ventilator weaning. A recent prospective randomized study compared the effects of dexmedetomidine or midazolam infusion together with an alfentanil infusion for analgesia if required on the inflammatory responses and gastric intramucosal pH in critically ill patients [111]. Fourty patients were included, and there was no statistically significant differences between the groups with respect to hemodynamic and biochemical measurements, or gastric intramucosal pH. However, there were significant decreases in TNF-α, IL-1β, IL-6 at 24 hours in the dexmedetomidine group compared with the midazolam group. Another recent prospective double-blind randomized study compared the efficacy and safety of prolonged sedation with dexmedetomidine and midazolam among 375 mechanically ventilated patients [148]. Infection rate was significantly lower in the dexmedetomidine group compared with the midazolam group (10.2 vs 19.7%, *P *= 0.02). Although length of ICU stay was similar in the two groups, median time to extubation was significantly shorter in the dexmedetomidine group compared with the midazolam group (3.7 vs 5.6 days, *P *= 0.01).

A retrospective study compared the rate of pneumonia between ventilated head trauma patients who received thiopental therapy (n = 75) and those who did not receive thiopental (n = 76) [149]. The rate of noscomial pneumonia was higher in patients who received thiopental compared with those who did not receive thiopental (53 vs 35%; OR, 1.85; 95% CI, 0.97 to 3.51). In addition, thiopental therapy was independently associated with nosocomial pneumonia. Results of studies comparing different sedative agents with regard to cytokine levels, infection rate and other outcomes are presented in Table 5.

**Table 5:** Results of clinical studies comparing different sedative agents with regard to cytokine levels, infection rate, and duration of mechanical ventilation

Outcome	First author [Reference]	Year of publication/country	Study design/Number of patients	Main results*
Cytokine responses	von Dossow [143]	2008/Germany	Randomized controlled study comparing fentanyl with remifentanil/40 patients	IFNγ/IL-10 after concanavalin A stimulation, and SOCS-3 gene expression significantly lower in remifentanil group
	Helmy [145]	2001/Egypt	Randomized controlled study comparing propofol with midazolam/40 patients	Both agents suppressed IL-8 production Midazolam suppressed production of IL-1β, IL-6, and TNF-α Propofol inhibited IL-2 production and stimulated IFNγ production
	Memis [111]	2007/Turkey	Randomized controlled study comparing dexmedetomidine vs midazolam/40 patients	Significant decreases in TNF-α, IL-1β, and IL-6 in dexmedetomidine group
Infection and other outcomes	Arya [144]	2001/India	Randomized controlled study comparing midazolam and morphine with midazolam/33 newborn babies	Comparable rate of infection (6%) in the two groups
	Muellejans [14]	2006/Germany	Randomized controlled study comparing remifentanil and propofol with fentanyl and midazolam/80 patients	Mean time intervals from arrival at the ICU until extubation (20.7 vs 24.2 hours) and from arrival until eligible discharge from the ICU (46.1 vs 62.4 hours) were significantly (*P *< 0.05) shorter in the remifentanil/propofol group
	Rozendaal [15]	2009/Neatherlands	Randomized controlled study comparing remifentanil and propofol with propofol, midazolam or lorazepam combined with fentanyl or morphine/215 patients	The remifentanil-based regimen reduced median weaning time by 18.9 hours (*P *= 0.0001), increased the likelihood to be extubated (*P *= 0.018), and the discharge from the ICU (*P *= 0.05)
	Kress [146]	1996/USA	Randomized controlled study comparing propofol with midazolam/73 patients	Narrower range of wake-up times with a higher likelihood of waking in less than 60 minutes in propofol group
	Riker [148]	2009/USA	Randomized controlled double-blind study comparing dexmedetomidine with midazolam/375 patients	Reduced rate of infection (10.2 vs 19.7%, *P *= 0.02), and shorter time to extunation (median 3.7 vs 5.6 days, *P *= 0.01) in the dexmedetomidine group
	Nadal [149]	1995/Spain	Retrospective cohort comparing patients with thiopental with those without thiopenthal	Higher rate of VAP in patients who received thiopenthal (53 vs 35%)

ICU: intensive care unit; IFN: interferon; IL: interleukin; TNF: tumour necrosis factor; VAP: ventilator-associated pneumonia.

#### Limitations

Our review has some limitations. First, there is strong evidence coming from animal studies that sedative agents could alter immune function and increase the risk of infection. However, clinical studies are needed to determine whether these data are relevant in the clinical setting. The epidemiologic studies showed a link between sedation and infection. However, no cause-to-effect relation could be demonstrated. Second, the subject of our review is vast and the literature covering the effects of sedative agents on immune function is very large. Therefore, this could not be a comprehensive review of the total literature on this subject within the size of the article. Third, some sedative agents used for short sedation, such as etomidate, were not reviewed. In addition, effects of muscle relaxants on infection were not reviewed.

#### Future studies

Future studies should compare the effect of different sedative agents on the incidence of ICU-acquired infection. Further, the impact of progressive opioid discontinuation on the risk of ICU-acquired infection should be compared with abrupt discontinuation. The role of intermittent dosing rather than infusion of sedative agents should also be evaluated. The impact of adjunctive agents, such as clonidine, should be evaluated. In addition, analgesics other than opioids should be explored in ICU patients, and the risk of ICU-acquired infections should be compared between opioids and other analgesics. Volatile sedation using isoflurane appears a promising alternative to intravenous sedatives for adult patients mechanically ventilated in the ICU. Finally, peripherally acting mu-opioid receptor antagonists methylnatrexone and alvimopan are a new class of drugs designed to reverse opioid-induced side effects on the gastrointestinal system without compromising pain relief [150]. A recent randomized controlled study demonstrated that methylnatrexone rapidly induced laxation in patients with advanced illness and opioid-induced constipation [151]. Treatment did not appear to affect central analgesia or precipitate opioid withdrawal. Future studies should determine whether these results are applicable in ICU patients, and whether treatment with these antagonists could influence gastrointestinal translocation and ICU-acquired infections.

## Conclusions

Sedation is associated with increased risk of ICU-acquired infection. Prolongation of exposure to risk factors for infection, microaspiration, gastrointestinal motility disturbances, microcirculatory effects, and immunomodulatory effects are the main mechanisms by which sedation might favor infection in critically ill patients. Clinical studies comparing different sedative agents do not provide evidence to recommend the use of a particular agent to reduce ICU-acquired infection rate. However, sedation strategies aiming to reduce the duration of mechanical ventilation, such as daily interruption of sedatives or nursing-implementing sedation protocol, should be promoted. In addition, the use of short-acting opioids, propofol, and dexmedetomidine is associated with shorter duration of mechanical ventilation and ICU stay, and might be helpful in reducing ICU-acquired infection rates.

## Key messages

• Several epidemiologic studies suggest a link between sedation and ICU-acquired infection.

• Prolongation of exposure to risk factors for infection, microaspiration, gastrointestinal motility disturbances, microcirculatory effects and immunomodulatory effects are main mechanisms by which sedation may favor infection in critically ill patients.

• Clinical studies comparing different sedative agents do not provide evidence to recommend the use of a particular agent to reduce ICU-acquired infection rate.

• Sedation strategies aiming to reduce the duration of mechanical ventilation, such as daily interruption of sedatives or nursing-implementing sedation protocol, should be promoted.

• The use of short-acting opioids, propofol, and dexmedetomidine is associated with shorter duration of mechanical ventilation and ICU stay, and might be helpful in preventing ICU-acquired infections.

## Abbreviations

CI: confidence interval; CLP: cecal ligation and puncture; Con: concanavalin; GMP: guanosine monophosphate; HCV: hepatitis C virus; ICU: intensive care unit; IL: interleukin; IFN: interferon; IQR: interquartile range; LPS: liposaccharide; NK: natural killer; NO: nitric oxide; OR: odds ratio; PBR: peripheral benzodiazepines receptor; SIV: simian immunodeficiency virus; SOCS: suppressor of cytokine signalling; TNF: tumor necrosis factor; TSST: toxic shock syndrome toxin; VAP: ventilator-associated pneumonia.

## Competing interests

The authors declare that they have no competing interests.

## Authors' contributions

SN, DeM, DaM, AD, and CHM designed this review. SN, and DeM collected data. SN wrote the manuscript, and all authors participated in its critical revision. SN had full access to all data in the study and had final responsibility for the decision to submit for publication. All authors read and approved the final manuscript.
